# Coupling Kinesin Spindle Protein and Aurora B Inhibition with Apoptosis Induction Enhances Oral Cancer Cell Killing

**DOI:** 10.3390/cancers16112014

**Published:** 2024-05-25

**Authors:** João P. N. Silva, Bárbara Pinto, Luís Monteiro, Patrícia M. A. Silva, Hassan Bousbaa

**Affiliations:** 1 UNIPRO—Oral Pathology and Rehabilitation Research Unit, University Institute of Health Sciences (IUCS), Cooperativa de Ensino Superior Politécnico e Universitário (CESPU), Rua Central de Gandra, 1317, 4585-116 Gandra, Portugal; joaosilva_06@hotmail.com (J.P.N.S.); barbara_fernandes_pinto@hotmail.com (B.P.); luis.monteiro@iucs.cespu.pt (L.M.); 2 Associate Laboratory i4HB, Institute for Health and Bioeconomy, University Institute of Health Sciences-CESPU, 4585-116 Gandra, Portugal; 3 UCIBIO—Applied Molecular Biosciences Unit, Translational Toxicology Research Laboratory, University Institute of Health Sciences (1H-TOXRUN, IUCS-CESPU), 4585-116 Gandra, Portugal

**Keywords:** KSP inhibitor, Aurora B inhibitor, Navitoclax, oral cancer, antimitotics, combination treatment

## Abstract

**Simple Summary:**

Scientists are studying proteins like kinesin spindle protein and Aurora B, crucial for cell division and potential targets in cancer treatment. Drugs aimed at these proteins show promise in lab tests for killing cancer cells, but in clinical trials alone, they are not always effective, possibly due to varied cancer cell responses. To enhance their efficacy, researchers are exploring combinations with other cell-killing drugs. Our study focused on Navitoclax, an inducer of cancer cell death, tested alongside Ispinesib and Barasertib, targeting kinesin spindle protein and Aurora B, respectively. Together, these drugs induced significant cancer cell death, mainly through apoptosis. Moreover, imaging techniques revealing their combined effects suggest that combining these drugs could be a potent cancer treatment strategy, warranting further investigation in clinical trials.

**Abstract:**

Many proteins regulating mitosis have emerged as targets for cancer therapy, including the kinesin spindle protein (KSP) and Aurora kinase B (AurB). KSP is crucial for proper spindle pole separation during mitosis, while AurB plays roles in chromosome segregation and cytokinesis. Agents targeting KSP and AurB selectively affect dividing cells and have shown significant activity in vitro. However, these drugs, despite advancing to clinical trials, often yield unsatisfactory outcomes as monotherapy, likely due to variable responses driven by cyclin B degradation and apoptosis signal accumulation networks. Accumulated data suggest that combining emerging antimitotics with various cytostatic drugs can enhance tumor-killing effects compared to monotherapy. Here, we investigated the impact of inhibiting anti-apoptotic signals with the BH3-mimetic Navitoclax in oral cancer cells treated with the selective KSP inhibitor, Ispinesib, or AurB inhibitor, Barasertib, aiming to potentiate cell death. The combination of BH3-mimetics with both KSP and AurB inhibitors synergistically induced substantial cell death, primarily through apoptosis. A mechanistic analysis underlying this synergistic activity, undertaken by live-cell imaging, is presented. Our data underscore the importance of combining BH3-mimetics with antimitotics in clinical trials to maximize their effectiveness.

## 1. Introduction

Cancer of the oral cavity and lip is the most common type of head and neck cancer with 377,713 new cases and 177,757 deaths reported in 2020 according to GLOBOCAN [1]. Approximately 90% of all oral cancers are oral squamous cell carcinomas (OSCCs) [2]. The most common origin sites for OSCC are the tongue and the floor of the mouth [2,3]. The emergence of this type of malignancy is mainly associated with tobacco and alcohol consumption. Nonetheless, other factors such as human papillomavirus infection can also lead to OSCC development [4,5]. Despite the different therapeutic strategies, small improvements in the treatment of oral cancer have been reported and high mortality rates for advanced disease are still observed [6]. Thus, new treatment options are needed.

Combinatorial approaches have risen as a hallmark in the treatment of cancer since they can lead to prolonged responses with lower toxicity and potential synergistic effects [7]. Microtubule-targeting agents (MTAs) have been extensively explored and some, such as paclitaxel and docetaxel, have been approved and are used for the treatment of head and neck squamous cell carcinoma (HNSCC) mostly in combination with platinum, 5-fluorouracil, and cetuximab [8]. Nonetheless, MTAs are associated with some disadvantages, including high toxicity due to low specificity and therapeutic resistance [9]. To overcome these issues, drugs that target specific proteins involved in mitosis, known as antimitotic agents, such as kinesins, like kinesin spindle protein (KSP), and kinases, like Aurora B, have been investigated [10].

KSP, also known as Eg5 and Kif11, is a kinesin-5 family member essential for bipolar mitotic spindle formation, microtubule cross-linking, and chromosome alignment [11,12,13]. The overexpression of KSP is associated with poor outcomes in breast and laryngeal cancers and its inhibition leads to the formation of monopolar spindles, activation of the spindle assembly checkpoint (SAC), and consequently, mitotic arrest followed by cell death [11,14].

Aurora B is a part of the Aurora kinase family that includes Aurora A and Aurora C and it plays a role in chromosome segregation and cytokinesis [15]. The overexpression of Aurora B was reported for metastatic and poorly differentiated OSCC suggesting this kinase is involved in OSCC progression [16]. In addition, Aurora B inhibition can lead to polyploidy, and consequently cell death [15,17].

Even though the inhibition of these proteins in preclinical trials showed promising results, their inhibitors have shown disappointing results as monotherapy in clinical trials, due mainly to the lack of efficacy [18,19,20,21,22]. Different possibilities have been suggested for this lack of efficacy. For instance, the fact that antimitotics act only during mitosis leads to low efficacy due to the administration schedules since only a low fraction of tumor cells will be undergoing mitosis at any given point in time. Additionally, mitotic slippage, a phenomenon wherein cells exit mitosis without division, culminating in aneuploidy and fostering cancer cell survival, has also been pointed as one of the major causes of antimitotic treatment resistance [23]. According to a recently proposed model, cell fate during mitotic arrest is defined by the duration of SAC activity, level of BCL-xL, and cyclin B1 degradation [24]. In this model, the increased duration of SAC activity enhances the probability of cell death. The level of BCL-xL also plays an important role. For instance, when cells are arrested in mitosis, the apoptotic signaling threshold is reached when cells present the low activity or inhibition of BCL-xL, leading to death in mitosis. On the contrary, high levels of BCL-xL allow the cyclin B1 degradation threshold to be reached, and mitotic slippage occurs. Post-slippage death occurs when the level of BCL-xL is high enough to allow mitotic exit but not enough to block the apoptotic threshold to be reached. BCL-xL is a prosurvival protein involved in the suppression of the intrinsic apoptotic pathway through the inhibition of cytochrome-c release from mitochondria, and its overexpression in OSCC is associated with poor prognosis [25]. Apoptosis is an important mechanism to prevent cancer development since it reduces the risk of genomic instability occurrence [26,27]. However, cancer cells acquire resistance to cell death by overexpressing prosurvival proteins, including BCL-2, BCL-xL, and BCL-w, while downregulating pro-apoptotic ones [28]. Thus, the addition of an inhibitor of BCL-xL to antimitotics should increase the apoptotic signaling and lead to increased cell death in mitosis preventing mitotic slippage or increasing post-slippage death. Accordingly, in several types of cancer cells, it was shown that the combination of the inhibitors of the prosurvival BCL-2 family members with antimitotics enhanced their efficacy by promoting apoptosis [29,30,31,32].

Therefore, in this work, we analyzed the effects of the addition of Navitoclax, an BCL-2 and BCL-xL inhibitor, with Ispinesib, a KSP inhibitor, or Barasertib, an Aurora B inhibitor, on oral cancer cells and showed that it enhanced the therapeutic potential of KSP and Aurora B targeting by increasing cell death during mitosis or post-slippage, respectively.

## 2. Materials and Methods

### 2.1. Small Molecule Inhibitors

The inhibitors of KSP (Ispinesib and Filanesib), Aurora B (Barasertib and SP-96), and BCL-2/BCL-xL (Navitoclax and ABT-737) were obtained from MedChem Express (Shanghai, China). Stock concentrations of 5 or 10 mM were prepared by resuspending the inhibitors in sterile dimethyl sulfoxide (DMSO, Sigma-Aldrich Co., Ltd., St. Louis, MO, USA). Multiple aliquots were made and stored at −20 °C to avoid the necessity of repeated freezing and thawing cycles. To mitigate potential DMSO toxicity, the working solutions were prepared in a fresh medium and used to make solutions with the desired concentrations.

### 2.2. Cell Culture

SCC25 (tongue squamous cell carcinoma; The Global Bioresource Center-ATCC**^®^** CRL-1628) and SCC09 (tongue squamous cell carcinoma; The Global Bioresource Center-ATCC**^®^** CRL-1629) tumor cell lines were grown in DMEM/F12 culture medium (Dulbecco’s Modified Eagle Medium/Nutrient Mixture F-12, PAN-Biotech, Aidenbach, Germany), supplemented with 10% of the heat-inactivated fetal bovine serum (FBS, Biochrom, Berlin, Germany) and 1% of Pen/Strep (Biochrom). The non-tumor cell line HOK (human oral keratinocyte, ScienCell Research Laboratories, Carlsbad, CA, USA) was grown in OKM culture medium (oral keratinocyte medium, Innoprot, Bizkaia, Spain). The cell lines were kept at 37 °C with 5% CO_2_ in an incubator (Hera Cell, Heraeus, Hanau, Germany) while ensuring humidity was maintained.

### 2.3. RNA Extraction, cDNA Synthesis, and Real-Time PCR

Total RNA was extracted from the cell lines HOK, SCC09, and SCC25, and cDNA was synthetized as previously described [33]. iQ™ SYBR Green Supermix Kit (Bio-Rad, Laboratories, Inc., Hercules, CA, USA) was used for DNA amplification on an iQ Thermal Cycler (Bio-Rad) using the following program: initial denaturing step at 95.0 °C for 3 min; 38 cycles at 94.0 °C for 20 s; 60.0 °C for 30 s and 72.0 °C for 30 s. The melting curve encompassed temperatures ranging from 65.0 to 95.0 °C, with 0.5 °C increments for 5 s each. The primers used, at a concentration of 10 µM, were as follows: KSP: forward 5′-GAACAATCATTAGCAGCAGAA-3′ and reverse 5′-TCAGTATAGACACCACAGTTG-3′; Aurora B: forward 5′-AGAAGGAGAACTCCTACCCCT-3′ and reverse 5′-CGCGTTAAGATGTCGGGTG-3′; 18S: forward 5′-CAACATCGATGGGCGGCGGA-3′ and reverse 5′-CCCGCCCTCTTGGTGAGGTC-3′; GAPDH: forward 5′-ACAGTCAGCCGCATCTTC-3′ and reverse 5′-GCCCAATACGACCAAATCC-3′; Actin: forward 5′-AATCTGGCACCACACCTTCTA-3′ and reverse 5′-ATAGCACAGCCTGGATAGCAA-3′. The data were analyzed using the CFX ManagerTM Software (version 1.0, BioRad), and the relative quantification was calculated using the ΔΔCT method. The data were normalized against the housekeeping genes Actin and 18S for KSP and Actin and GAPDH for Aurora B.

### 2.4. Protein Extraction and Western Blotting

The HOK, SCC09, and SCC25 cell line total protein extraction was performed by first centrifuging the cells and then resuspending them in a lysis buffer containing 50 mM Tris pH 7.5, 150 mM NaCl, 1 mM EDTA, and 1% Triton-100, supplemented with a protease inhibitor cocktail (Sigma–Aldrich (St. Louis, MO, USA)). The proteins were then quantified by using the BCA^TM^ Protein Assay Kit (Pierce Biotechnology (Rockford, IL, USA)) according to the manufacturer’s instructions. Subsequently, 15 µg of protein lysate was resuspended with SDS-sample buffer consisting of 375 mM Tris pH 6.8, 12% SDS, 60% Glycerol, 0.12% Bromophenol Blue, and 600 nM DTT, and denatured for 3 min at 95 °C. The proteins were then separated using the SDS–PAGE gels of 7.5% to resolve high molecular weight proteins such as KSP (120–130 kDa), and 10% to resolve low molecular weight proteins such as Aurora B (39–45 kDa). Protein transfer from the gels onto nitrocellulose membranes (Amersham (Staffordshire, UK)) was carried out using the Trans-Blot Turbo Transfer System from Bio-Rad. Afterwards, 5% non-fat dried milk in TBST (50 mM Tris pH 7.5, 150 mM NaCl, 0.05% Tween-20) was used to block the membranes. Then, the membranes were incubated overnight at 4 °C with the following primary antibodies diluted in TBST: mouse anti-α-tubulin (1:5000, T568 Clone B-5-1-2, Sigma–Aldrich), rabbit anti-KSP (1:1000, abcam), and rabbit anti-Aurora B (1:1000, Sigma-Aldrich). After washing the membranes with TBST containing 1% skim milk three times for 5 min each, they were incubated with suitable horseradish peroxidase-conjugated secondary antibodies (1:1500, Vector) for 1 h at room temperature. Protein detection was carried out using the Enhanced Chemiluminescence (ECL) method with a ChemiDOc system (Bio-Rad). Protein signal intensity was quantified using the Image Lab 6.1v software. The normalization of protein values was performed using the expression levels of α-tubulin.

### 2.5. Indirect Immunofluorescence

The SCC25 cells were seeded at a density of 0.1 × 10^6^ cells/mL on poly-L-lysine-coated coverslips in a complete culture medium for 24 h. Following this, the cells were treated with 1.875 nM Ispinesib, 1000 nM Barasertib, as well as 3000 nM Navitoclax. After 24 h, the cells were fixed with methanol (Sigma-Aldrich, Co., Ltd., Gillingham, UK) at −20 °C for 10 min and then washed three times with PBS for 5 min each. Subsequently, the cells were blocked with 10% FBS in PBST (0.05% Tween-20 in PBS) for 30 min at room temperature, followed by a 1 h incubation with primary antibodies (mouse anti-α-tubulin, 1:2500, Sigma-Aldrich Co., Ltd., Gillingham, UK; human anti-CREST [34], 1:4000, gift from E. Bronze-da-Rocha, University of Porto, Portugal) diluted in PBST with 5% FBS. After three washes with PBST, the cells were incubated with Alexa Fluor 488- and 568-conjugated secondary antibodies (1:1500, Molecular Probes, Eugene, OR, USA). The staining of DNA was accomplished using 2 µg/mL of 4′,6-diamidino-2-phenylindole (DAPI, Sigma-Aldrich) diluted in Vectashield mounting medium (Vector, H-1000, Burlingame, CA, USA).

### 2.6. MTT Assay

To evaluate cell viability, a cytotoxicity assay using the tetrazolium salt 3-(4,5-dimethylthiazol-2-yl)-2,5-diphenyltetrazolium bromide (MTT) was performed. For seeding, 0.05 × 10^6^ SCC25 cells/mL and 0.1 × 10^6^ SCC09 cells/mL were plated in 96-well plates. The cells were allowed to adhere to the wells for 24 h with only medium. Subsequently, the culture medium was replaced with fresh medium containing the 2-fold serial dilutions of the inhibitors: 1.875 nM to 30 nM for Ispinesib, 1000 nM to 16,000 nM for Barasertib, 1500 nM to 24,000 nM for Navitoclax, 0.9375 nM to 15 nM for Filanesib, 1000 nM to 16,000 nM for SP-96, and 1500 nM to 24,000 nM for ABT-737 for SCC25 cells, and 3.75 nM to 60 nM for Ispinesib, 4000 nM to 64,000 nM for Barasertib, and 1000 nM to 16,000 nM for Navitoclax for SCC09 cells. After 48 h of exposure, 200 µL of non-supplemented medium and 20 µL of MTT tetrazolium salt solution (5 mg/mL PBS) were added to each well, followed by incubation for 2–4 h at 37 °C. Subsequently, the medium was aspirated, and 100 µL of DMSO was added to dissolve the resulting formazan crystals. Optical density was measured at 570 nm using a microplate reader (Biotek Synergy 2, Winooski, VT, USA) equipped with the Gen5 software (version 1.07.5, Biotek, Winooski, VT, USA). The IC_50_ values were calculated using GraphPad Prism version 8 (GraphPad Software Inc., San Diego, CA, USA) using the nonlinear regression analysis. The effects of combinations were evaluated using the Combenefit Software (version 2.021, Cancer Research UK Cambridge Institute, Cambridge, UK) with a dual-drug crosswise concentration analysis.

### 2.7. Apoptosis Detection Using Annexin V/PI Staining

To evaluate apoptotic cell death, the Annexin V-FITC Apoptosis Detection Kit (eBioscience, Vienna, Austria) was employed according to the manufacturer’s guidelines. Briefly, the SCC25 cells at a concentration of 0.1 × 10^6^ cells/mL were seeded into 6-well plates and, after 24 h, treated with Ispinesib and Barasertib alone or in combination with Navitoclax or Filanesib and SP-96 alone or in combination with ABT-737 at synergistic concentrations. Following a 48 h incubation period, the cells were harvested, centrifuged at 1000 rpm for 5 min, and suspended in 1× binding buffer. Annexin V-FITC was then added and incubated at room temperature for 10 min in the dark. After washing, the cells were suspended in 1x binding buffer, and Propidium Iodide (PI) at a concentration of 20 μg/mL was added. A BD Accuri™ C6 Plus Flow cytometer (BD Biosciences, Qume Drive, San Jose, CA, USA) was used to measure the fluorescence. The data were then analyzed using the BD Accuri TM C6 Plus software, version 1.0.27.1. The sample processing followed the manufacturer’s instructions for the “Annexin V-FITC Apoptosis Detection Kit”, with a minimum of 20,000 events collected per sample.

### 2.8. Mitotic Index Determination

In total, 0.1 × 10^6^ SCC25 cells were seeded in six-well dishes. After 24 h, Ispinesib alone or combined with Navitoclax or Filanesib alone or in combination with ABT-737 at the synergistic point concentrations were added. Positive controls for antimitotic activity were established by treating the cells with 1 µM of the microtubule depolymerizing agent nocodazole. The control groups included untreated cells and cells treated with DMSO, serving to evaluate solvent-induced cytotoxicity. The mitotic index, calculated as the percentage of mitotic cells within the overall cell population, was determined through the observation of cell rounding under phase-contrast microscopy after the 24 h treatment from ten random microscope fields.

### 2.9. Time-Lapse Microscopy

In total, 0.08 × 10^6^ SCC25 cells were seeded onto LabTek II chambered cover glass (Nunc, Penfield, NY, USA). The remaining wells were filled with sterile water to maintain a humidified atmosphere. Following overnight incubation at 37 °C under 5% CO_2_, the medium was replaced with fresh medium containing Ispinesib/Barasertib alone or in combination with Navitoclax at synergistic concentrations. To capture time-lapse images at 5 min intervals over 48 h, an Axio Observer Z.1 SD inverted microscope (Carl Zeiss (Oberkochen, Germany)) equipped with an incubation chamber set to 37 °C and 5% CO_2_ using differential interference contrast (DIC) optics and a 63× objective was used. Time-lapse image sequences were compiled into movies using the ImageJ software (version 1.47, Rasband (New York, NY, USA), W.S., ImageJ, U. S. National Institutes of Health, Bethesda, MD, USA).

### 2.10. Phase-Contrast and Fluorescence Microscopy Images

Phase-contrast microscopy images were obtained using a Nikon TE 2000-U microscope (Nikon, Amsterdam, The Netherlands) with a 10× objective, connected to a DXM1200F digital camera controlled by Nikon ACT-1 software version 2.63 (Melville, NY, USA). An Axio Observer Z.1 SD microscope (Carl Zeiss, Jena, Germany) with an AxioCam MR3 equipped with a Plan Apochromatic 63×/NA 1.4 objective was used to capture fluorescence images. ImageJ version 1.47 was used to process fluorescence images.

### 2.11. Colony Formation Assay

A total of 850 SCC25 cells were seeded in six-well plates. After 24 h of incubation, Ispinesib or Barasertib alone or in combination with Navitoclax, at the respective synergistic point concentrations, were added. The control groups included untreated cells and cells treated with DMSO. After 48 h, the medium was removed and DMEM F12 medium without drugs was added. The cells were then incubated for a duration of 6 days. Next, colony fixation was performed by the addition of 100% methanol at −20 °C for 20 min. The staining was performed by the addition of violet crystal (Merck) 0.05% (*w*/*v*) in distilled water for 30 min. At least three independent experiments were used to obtain the number of colonies for each condition. Plating efficiency (PE) was determined by calculating the percentage of grown colonies over the number of cells seeded in the control. The survival fraction for each condition was then calculated as the ratio of the number of colonies to the number of cells seeded, multiplied by 1/PE.

### 2.12. Statistical Analysis

All experiments were conducted in triplicate, and a minimum of three independent experiments were performed. The data are expressed as mean ± standard deviation (SD). Statistical analyses were conducted using GraphPad Prism Software Inc. (La Jolla, CA, USA) v8, employing one-way or two-way ANOVA with Tukey’s multiple comparison test. The values of * *p* < 0.05, ** *p* < 0.01, *** *p* < 0.001, and **** *p* < 0.0001 were deemed statistically significant.

## 3. Results

### 3.1. KSP and Aurora B Proteins Are Overexpressed in Oral Squamous Carcinoma Cells

KSP is a plus-end directed kinesin essential for bipolar spindle formation, and it was suggested that its overexpression in mice can lead to genomic instability and tumor development [12,14]. Its inhibition leads to the formation of monopolar spindles, activation of SAC, and consequently, mitotic arrest typically followed by cell death [11,14]. While Aurora B is a serine-threonine protein kinase member of the Aurora kinases family involved in correct chromosomal segregation and was shown to be overexpressed in poorly differentiated and metastasized OSCC [16,35]. Aurora B inhibition can lead to polyploidy, and consequently cell death [15,36].

**Figure 1:**
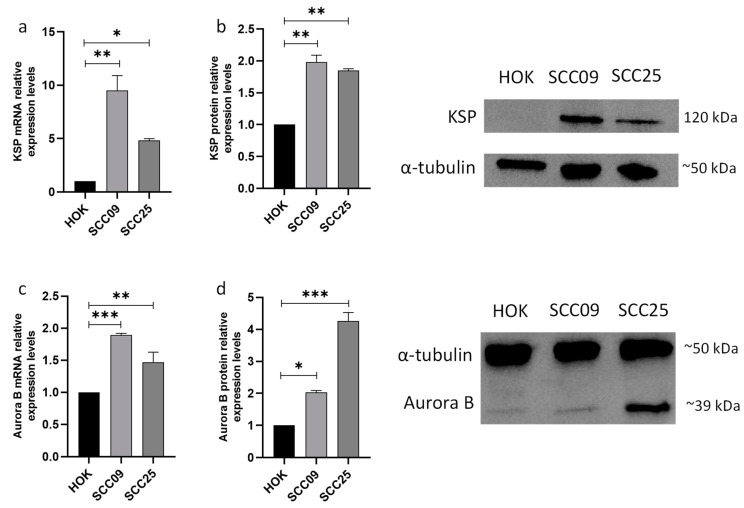
KSP and Aurora B show increased expression in oral squamous carcinoma cell lines. The mRNA expression levels of KSP (**a**) and Aurora B (**c**) were assessed through qRT-PCR in the oral cancer cell lines SCC09 and SCC25 and compared to the non-tumor human oral keratinocyte (HOK) cells. The quantification of the protein levels of KSP (**b**, left) and Aurora B (**d**, left) was performed by the Western blotting assay, with the protein α-tubulin as control. The representative Western blot images for KSP (**b**, right) and Aurora B (**d**, right) are presented. The data presented indicate the mean value along with the standard deviation (mean ± SD) obtained from three independent experiments. Statistical analysis was performed using one-way ANOVA followed by Tukey’s multiple comparisons test. The significance levels were as follows: * for *p* < 0.05, ** for *p* < 0.01, and *** for *p* < 0.001. Original western blots are presented in File S1.

Thus, we analyzed the mRNA and protein expression levels of these targets in two squamous cell carcinoma (SCC) cell lines, SCC25 and SCC09, through qRT-PCR and Western blot, respectively. The results show that the mRNA levels of both proteins are overexpressed in SCC25 and SCC09 when compared to the non-tumoral cell HOK (Figure 1a,c). The KSP and Aurora B protein expression levels also demonstrated an increase in both cell lines (Figure 1b,d).

These results show that these proteins can be potential targets for the treatment of oral cancer.

Next, we proceeded to target KSP and Aurora B and searched for possible synergism in oral cancer killing when combined with apoptosis targeting.

### 3.2. The Combinatorial Approaches with Ispinesib and Navitoclax and Barasertib with Navitoclax Show Synergistic Effects in Oral Cancer Cells

**Figure 2:**
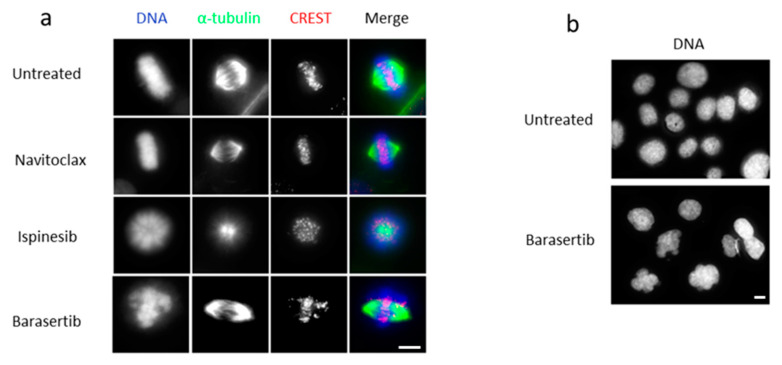
Mitotic defects induced by KSP and Aurora B inhibition. Illustrative immunofluorescence images showing SCC25 cells phenotype after 24 h treatment with 1.875 nM of Ispinesib or 1000 nM of Barasertib (**a**,**b**). DAPI was used to stain DNA (blue), while α-tubulin was stained to allow the visualization of microtubules (green), and CREST (red) for kinetochores localization. Bar, 5 μm.

Firstly, to confirm the specificity of the inhibitors under study, the phenotype of the cells treated with Ispinesib, Barasertib, and Navitoclax was assessed by immunofluorescence microscopy. All the drugs showed the expected phenotype with Ispinesib leading to a monopolar spindle phenotype in the cells arrested in mitosis, while the addition of Barasertib led to misalignment of chromosomes during metaphase and to a multinucleated phenotype after cell division (Figure 2a,b). The cells treated with Navitoclax maintained a bipolar spindle configuration.

**Figure 3:**
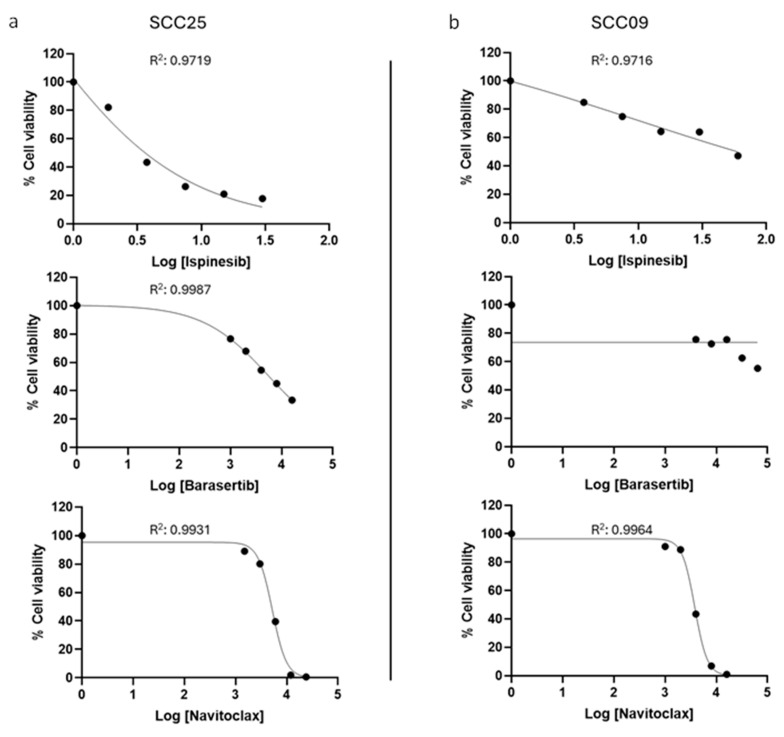
Dose−response curves of Ispinesib, Barasertib, and Navitoclax in SCC25 (**a**) and SCC09 (**b**) cell lines. The percentage of cell viability vs. the concentration of the different inhibitors (logarithmic scale) is shown. The R^2^ values are shown for each curve indicating the fit of the model to the data.

To determine the IC_50_ of Ispinesib, Barasertib, and Navitoclax along with their cytotoxicity both alone and in combination, the MTT assay was performed in the oral cancer cell lines SCC09 and SCC25 (Table 1) and the dose–response curves were obtained (Figure 3). We observed that the SCC25 cells were more sensitive to Ispinesib and Barasertib compared to the SCC09 cells, whereas both showed comparable sensitivity to Navitoclax.

**Figure 4:**
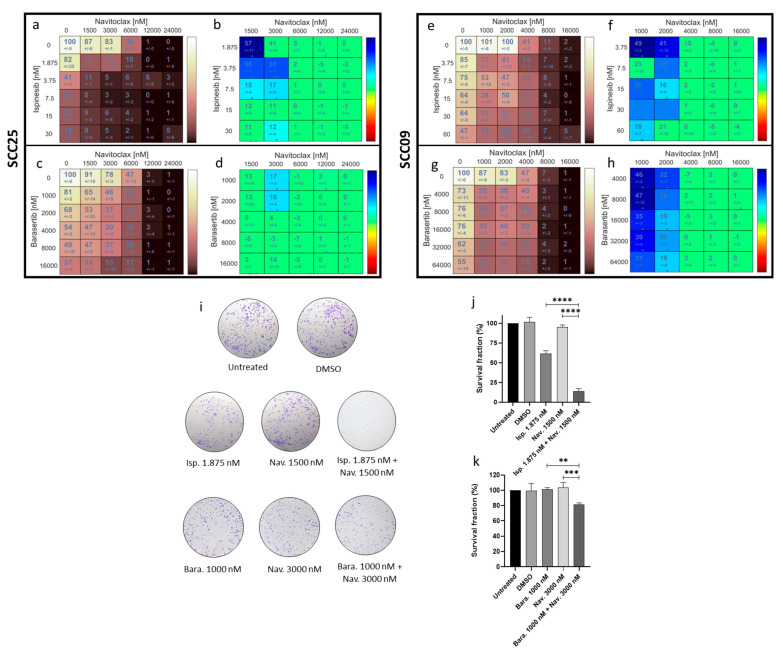
The combinatorial approaches Ispinesib + Navitoclax and Barasertib + Navitoclax enhance cytotoxicity in the SCC25 and SCC09 cell lines. Cell viability (%) following 48 h of drug exposure both alone or in combination (**a**,**c**,**e**,**g**), assessed by MTT assay with at least three independent experiments. The synergy scores were calculated using the Bliss model of the Combenefit software 2.021. Asterisks denote synergistic effects with statistical significance of * *p* < 0.05 and ** *p* < 0.01. (**b**,**d**,**f**,**h**). The representative images of colony formation assays following 6 days with SCC25 cells (**i**) are presented. The quantification of survival fraction (%) following treatment with drugs both alone and in combination is illustrated (**j**,**k**). The data presented are the average ± standard deviation of three separate experiments. Statistical analysis was performed using one-way ANOVA followed by Tukey’s post hoc test for multiple comparisons. The significance levels were as follows: ** for *p* < 0.01; *** for *p* < 0.001; and **** for *p* < 0.0001.

The viability assay data are presented as two dual-drug concentration crosswise matrices for each combination. Each matrix cell presents the percentage of viable cells for the drugs alone or in combination (Figure 4a,c,e,g) or the combinatorial interaction effect score (Figure 4,b,d,f,h). The results demonstrated that both combinations exhibited synergistic effects in both cell lines. To perform the subsequent experiments, we selected the SCC25 cell line since it displays the most favorable phenotypic characteristics for conducting microscopy assays. Furthermore, the concentrations used in the experiments for both combinations were the lower concentrations that showed synergistic effects (1.875 nM Ispinesib + 1500 nM of Navitoclax and 1000 nM Barasertib + 3000 nM of Navitoclax).

Moreover, to further assess if the synergistic effects observed were exclusive to these inhibitors or if they were due to the combinatorial approaches, we also used Filanesib, (another KSP inhibitor), SP-96 (another Aurora B inhibitor), and ABT-737 (another BCL-2 and BCL-xL inhibitor) for some of the experiments performed in this study. Filanesib and SP-96 synergized with ABT-737 and behaved similarly to the combinations of Ispinesib with Navitoclax and Barasertib with Navitoclax, respectively (Figure S1 and Table S1).

To analyze the long-term effects of these combinations in the proliferation of oral cancer cells, a colony formation assay was performed and the addition of Ispinesib alone led to nearly a 40% reduction in colony formation capacity (61.42 ± 3.83 %), while Barasertib showed no difference in the untreated cells (101.41 ± 2.13%). However, the addition of Navitoclax to both Ispinesib and Barasertib led to significant decreases in the survival fraction compared to the drugs administered alone (13.85 ± 3.40% and 81.18 ± 2.44%, respectively) (Figure 4i,j,k). These results suggest that the combinatorial approaches exhibit an ability to maintain long-term cellular cytotoxicity, preventing the proliferation of cancer cells.

Our data showed that BH3-mimetics synergize with both KSP and Aurora B inhibitors. Thus, we proceeded with further analyses of these combinations in order to gather a deeper comprehension of the mechanisms underlying these synergistic effects. We first began by analyzing the mechanisms underlying the synergistic cytotoxicity of the KSP inhibition plus Navitoclax combination in the next subsection; then we analyzed that of the Aurora B inhibition plus Navitoclax combination in the subsequent subsection.

### 3.3. The Combined Treatment with Ispinesib and Navitoclax Enhances Death in Mitosis in Oral Cancer Cells

When cells are arrested in mitosis, the two following outcomes can occur: death in mitosis or premature mitotic exit without undergoing cytokinesis, also known as mitotic slippage. Cell fate is decided according to the “competing networks-threshold model” where when cyclin B1 levels reach below the mitotic exit threshold first than apoptotic signaling reaches the apoptotic threshold, cells undergo mitotic slippage. On the other hand, if apoptotic signaling reaches the threshold first cell, death occurs [23]. Vorobjev et al. propose that when cells have a high concentration or high activity of BCL-xL, cyclin B1 degradation will reach the threshold for mitotic exit first before the apoptotic threshold is reached. However, post-slippage death (PSD) can occur if the level of BCL-xL is not high enough for cells to survive after mitotic exit. So, the level of the expression of BCL-xL at the time of slippage is the decisive factor between cell survival or PSD [24]. Therefore, with the purpose of promoting the apoptosis pathway, we analyzed the addition of Navitoclax to Ispinesib in oral cancer cells. To analyze the effect of this combination regarding mitotic index (MI) we used double the concentration of Ispinesib (3.75 nM) to ensure we would observe a clear outcome. Both the untreated cells (5.75 ± 0.46%) and the DMSO-treated (5.65 ± 0.42%) cells showed similar MIs showing no effect of the solvent at the concentration of 0.1%.

**Figure 5:**
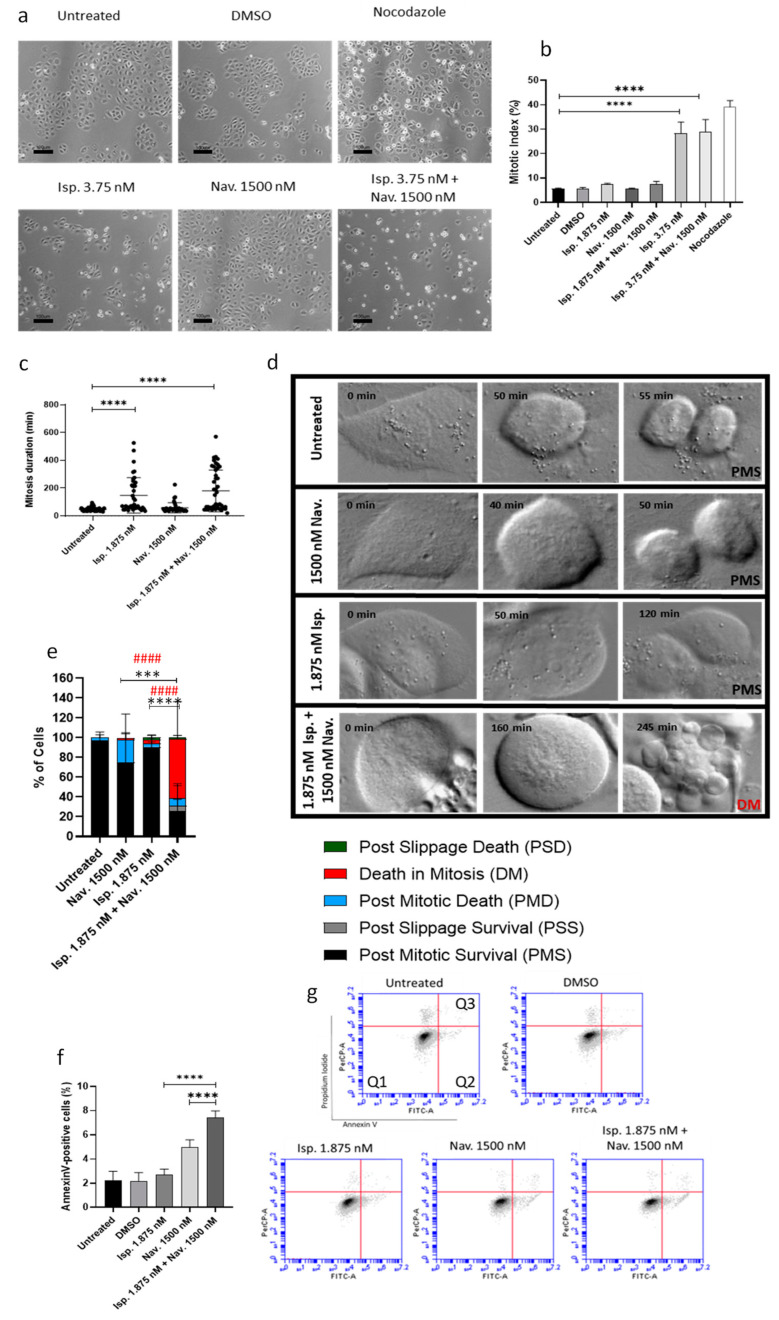
Addition of Navitoclax to Ispinesib increases cell death during mitosis in oral cancer cells. The representative images acquired by phase-contrast microscopy after drug exposure for 24 h for SCC25 (**a**). Mitotic index quantification for the SCC25 cell line (**b**); 0.2% of DMSO (drug solvent) was used as the negative control, while 1 μM of Nocodazole (mitotic blocker drug) was used as the positive control. The data presented are the average ± standard deviation of three separate experiments. Statistical analysis was performed using one-way ANOVA followed by Tukey’s post hoc test for multiple comparisons. **** *p* < 0.0001. The measurement of the duration of mitosis following the indicated drug treatments by time-lapse microscopy (**c**). The assessment of cell fate (%) over 48 h using indicated treatments (**f**). The representative time-lapse image sequences acquired during 48 h of exposure to drugs both alone and in combination (**d**). The assess-ment of cell fate (%) over 48 h using indicated treatments (**e**). The data presented are the average ± standard deviation of three separate experiments. Statistical analysis was performed using two-way ANOVA followed by Tukey’s post hoc test for multiple comparisons. #### (*p* < 0.0001) statistically significant difference in the cells that underwent death in mitosis (%) between 1500 nM Navitoclax or 1.875 nM Ispinesib and 1.875 nM Ispinesib + 1500 nM Navitoclax. *** (*p* < 0.001) postmitotic survival cell (%) difference between 1500 nM Navitoclax and 1.875 nM Ispinesib + 1500 nM Navitoclax. **** (*p* < 0.0001) postmitotic survival cell (%) difference between 1.875 nM Ispinesib and 1.875 nM Ispinesib + 1500 nM Navitoclax. The combination of Ispinesib and Navitoclax enhances cell death in the SCC25 oral cancer cell line. The quantification of Annexin-V-positive cells (**f**). Cytograms demonstrative of the oral cancer cells double stained with Annexin V-FITC and propidium iodide (PI) (**g**). The quadrants Q are defined as Q1 = living cells (Annexin V- and PI-negative), Q2 = early-stage apoptosis (Annexin V-positive/PI-negative), and Q3 = late-stage apoptosis/secondary necrosis (Annexin V- and PI-positive). The data presented are the average ± standard deviation of three separate experiments. Statistical analysis was performed using one-way ANOVA followed by Tukey’s post hoc test for multiple comparisons. The significance levels were as follows: **** for *p* < 0.0001.

In addition, the MI of the cells treated with Navitoclax (5.67 ± 0.21%) showed no significant difference from the untreated ones. Nonetheless, the addition of Ispinesib both alone (28.31 ± 4.57%) and in combination with Navitoclax (28.82 ± 5.18%) led to a similar increase in MI, compared to the untreated SCC25 cells (Figure 5a,b).

To further assess the effects of Ispinesib alone and in combination with Navitoclax regarding cell fate and mitotic duration, time-lapse microscopy was conducted.

The untreated cells underwent mitosis on average for 46.11 ± 13.77 min and the addition of Navitoclax (58.33 ± 37.51 min), similarly to MI, did not significantly affect this duration. On the other hand, the cells treated with Ispinesib showed a significant increase in the mitotic duration (147.18 ± 127.50 min) when compared with the untreated cells (Figure 5c). Furthermore, a similar duration to Ispinesib alone was observed for the combination with Navitoclax (179.44 ± 150.21 min). Regarding the cell fate, our results showed that the Navitoclax-treated cells underwent mostly normal cell cycling (75.09 ± 29.99%) with 24.91% of the cells dying mostly by postmitotic death (PMD) (22.83 ± 26.17%) (Video S1). Ispinesib alone showed a similar percentage of cells undergoing normal cell division (90.63 ± 5.20%) to the untreated cells (97.06 ± 5.88%), which suggests that the cells delayed in mitosis under Ispinesib at 1.875 nM manage to form functional spindles and undergo normal cell division (Video S2). When we looked at the effects of the combination of Ispinesib and Navitoclax, a significant reduction in postmitotic survival was observed, with only 25.48 ± 25.98% undergoing normal cell cycling, when compared to the untreated cells and cells treated with the inhibitors alone (Figure 5d,e; Video S3). In addition, the combination showed a significant increase in cell death (68.90%) of which 87.3% corresponds to death in mitosis (DM). These results showcase that the addition of Navitoclax to Ispinesib enhances cell death mostly during mitosis.

Since the combination showed increased cell death, our next step was to assess if it was attributable to the promotion of apoptosis by the addition of Navitoclax using flow cytometry. Our results show that the addition of Navitoclax slightly increases the percentage of apoptotic cells (4.99 ± 0.60%) while Ispinesib (2.68 ± 0.48%) showed no difference when compared to the control (2.3 ± 0.79%). Nonetheless, the combination of Ispinesib and Navitoclax significantly enhanced the apoptotic signaling (7.4 ± 0.59%) (Figure 5f,g).

These results demonstrate that the addition of Navitoclax to Ispinesib increases the apoptotic signaling of the cells arrested in mitosis leading to increased cell death, making this combination a promising approach that needs to be further explored.

### 3.4. Combining Barasertib-Mediated Aurora B Inhibition with Navitoclax Shifts the Cancer Cell Fate from Post-Slippage Cell Survival to Post-Slippage Cell Death

Aurora B plays a role in SAC activation by promoting mitotic checkpoint complex formation through the phosphorylation of Bub1 [17]. Consequently, inhibiting Aurora B suppresses sustained SAC activation [37]. In fact, the inhibition of Aurora B with Barasertib results in premature mitotic exit/mitotic slippage, ultimately leading to polyploidy [15,36]. According to Vorobjev et al.’s proposed model, the inhibition of BCL-xL and consequently increased apoptotic signaling should be enough to overcome mitotic slippage or lead to PSD. Thus, we proceeded to analyze the effects of the addition of Navitoclax to Barasertib.

**Figure 6:**
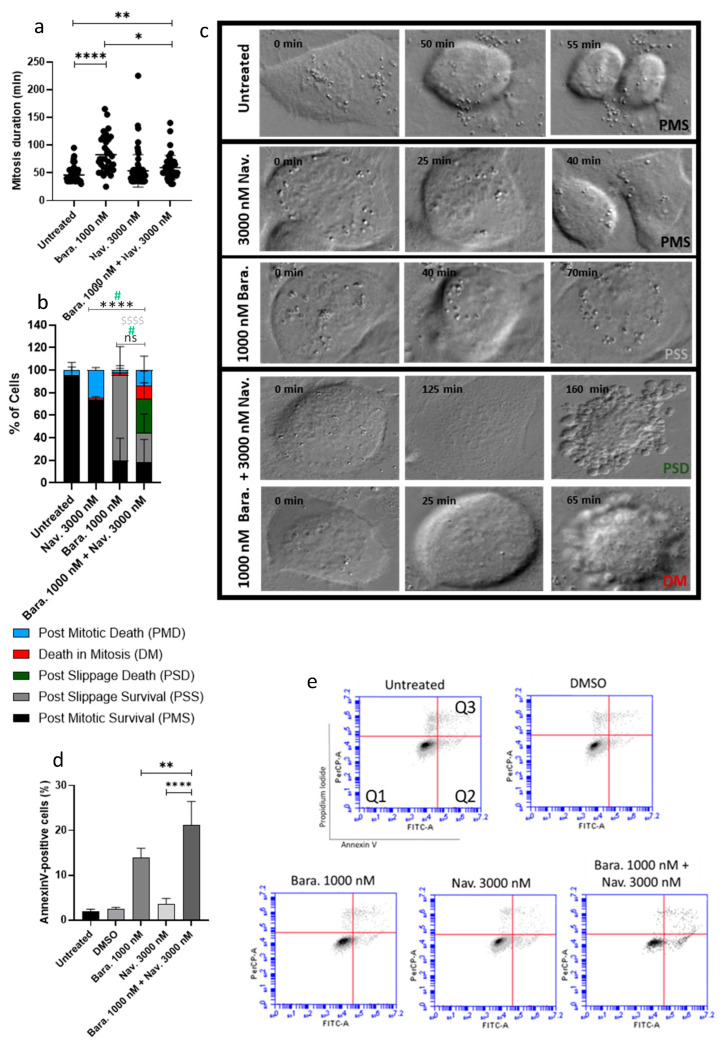
Addition of Navitoclax to Barasertib increases post-slippage death in oral cancer cells. The measurement of the duration of mitosis following the indicated drug treatments by time-lapse microscopy (**a**). The assessment of cell fate (%) over 48 h using indicated treatments (**b**). The representative time-lapse image sequences acquired during 48 h of exposure to drugs both alone and in combination (**c**). The data presented are the average ± standard deviation of three separate experiments. Statistical analysis was performed using two-way ANOVA followed by Tukey’s post hoc test for multiple comparisons. # (*p* < 0.05) statistically significant difference in the cells that underwent post-slippage death (%) between 3000 nM Navitoclax or 1000 nM Barasertib and 1000 nM Barasertib + 3000 nM Navitoclax. **** (*p* < 0.0001) postmitotic survival cell (%) difference between 3000 nM Navitoclax and 1000 nM Barasertib + 3000 nM Navitoclax. $$$$ (*p* < 0.0001) post-slippage survival cell (%) difference between 1000 nM Barasertib and 1000 nM Barasertib + 3000 nM Navitoclax. The addition of Barasertib to Navitoclax enhances cell death in the oral cancer cell line SCC25. The quantification of Annexin-V-positive cells (**d**). Cytograms demonstrative of the oral cancer cells double stained with Annexin V-FITC and propidium iodide (PI) (**e**). The quadrants Q are defined as Q1 = living cells (Annexin V- and PI-negative), Q2 = early-stage apoptosis (Annexin V-positive/PI-negative), and Q3 = late-stage apoptosis/secondary necrosis (Annexin V- and PI-positive). The data presented are the average ± standard deviation of three separate experiments. Statistical analysis was performed using one-way ANOVA followed by Tukey’s post hoc test for multiple comparisons. The significance levels were as follows: * for *p* < 0.05; ** for *p* < 0.01; and **** for *p* < 0.0001.

Firstly, we analyzed the cell fate and mitotic duration of the cells that underwent mitosis by time-lapse microscopy, and, as referred above, the untreated cells had on average a mitotic duration of 46.11 ± 13.77 min, and the treatment with Navitoclax did not significantly affect this duration (53.60 ± 29.25 min). In the oral cancer cell line SCC25, Barasertib alone nearly doubled the mitotic duration when compared with the untreated cells (82.88 ± 33.21 min) (Figure 6a). This was expected since Aurora B inhibition leads to a transient arrest before the cells prematurely exit mitosis by satisfying the SAC [38,39]. The addition of Navitoclax to Barasertib led to a significant decrease in the mitotic duration when compared to Barasertib alone (59.40 ± 22.04 min).

When assessing the cell fate, the addition of Navitoclax led to 74.26 ± 1.04% of the cells completing normal cycling while Barasertib alone showed a 95.55% cell survivability. However, of those cells, 79.51% underwent post-slippage survival (PSS) while only 20.49% experienced normal cell division. Nonetheless, the addition of Navitoclax to Barasertib led to a decrease in cell survival (44.17%) with most cells undergoing PSS (25.83 ± 17.02%) (Figure 6b,c). In addition, the combination led to increased cell death (55.83%) when compared with the untreated (2.94 ± 5.88%) and both drugs alone (4.44% and 25.74% for Barasertib and Navitoclax, respectively) groups. The enhanced cell death observed for the combination was mostly due to the promotion of PSD (30.83 ± 13.77%). In this sense, the addition of Navitoclax to Barasertib does not seem to greatly reduce the mitotic slippage of cells (75.97 vs. 56.66%, for Barasertib alone and in combination with Navitoclax, respectively) but increases substantially cell death mainly post-slippage.

To assess if increased cell death was attributable to increased apoptosis, the annexin V/propidium iodide analysis by flow cytometry was performed after 24 h exposure for the combination of Barasertib with Navitoclax. The cells were only exposed for 24 h to guarantee no cell undergoing apoptosis would be lost in the analysis since it led to the killing of a high number of cells plated for cytometry analysis at this time point. Our results showed no significant increase in apoptotic cells with the addition of Navitoclax (3.68 ± 1.17%) when compared to the control (2.03 ± 0.41%). Barasertib alone (13.98 ± 2.05%) led to an increased percentage of cells undergoing apoptosis while the combination with Navitoclax (21.13 ± 5.32%) exacerbated even further this increase (Figure 6d,e; Videos S4 and S5).

The findings indicate that inhibiting Aurora B leads to cell slippage following a brief delay in mitosis. These slipped cells managed to survive, at least for the duration of the experiment. However, when combined with Navitoclax, the slipped cells underwent cell death, mainly through apoptosis, indicating a suppression of anti-apoptotic signals that would otherwise support the survival of these cells. This further emphasizes the clinical importance of combining antimitotics with BH3-mimetics to enhance cancer cell death.

## 4. Discussion

Cancers of the oral cavity are the most common types of HNSCC, and their treatment consists mostly of surgery with or without radiotherapy and/or chemotherapy [40]. Even though an improvement in overall survival has been observed in recent years with a 5-year survival rate between 60% and 65%, patients with regional and distant metastases show lower rates (between 40% and 50% and less than 10%, respectively) [6]. Therefore, new therapeutic strategies are urgently needed. MTAs are widely used for the treatment of oral cancer but show several disadvantages such as high toxicity and lack of specificity [8,9]. Thus, drugs targeting specific proteins involved in mitosis, known as the second generation of antimitotics (SGAs), were developed. However, in clinical trials such as monotherapy, SGAs showed disappointing results [18,20]. In this sense, combinatorial approaches with SGAs should be explored to give these drugs a second chance. Thus, targeting two distinct pathways crucial for cancer cell viability through the combination of SGA drugs with apoptotic inducers could be a great alternative for anticancer strategy. The possible synergistic effects, the ability to target multiple pathways, the potential to overcome drug resistance, and the broad applicability make this type of combination an attractive approach for improving treatment outcomes in cancer patients.

The aim of this study was to assess the effects of combining a KSP inhibitor or an Aurora B inhibitor with an inhibitor of BCL-2 family prosurvival members in OSCC cell lines. In our work, we showed that KSP and Aurora B are overexpressed in both SCC09 and SCC25 cell lines which is in accordance with prior studies that showed that both proteins are overexpressed in oral cancer cell lines, making them potential targets for the treatment of oral cancer [16,41,42].

Interestingly, the addition of Ispinesib and Barasertib led to a higher IC_50_ in the SCC09 cell line regardless of protein expression. This may have been due to the fact that SCC25 has a higher proliferation rate than SCC09 with a doubling time of 2–3 days vs. 5–7 days, respectively, and consequently, antimitotics have more opportunities to promote cell death in SCC25 [43,44]. Furthermore, the SCC09 cell line seems to be resistant to Barasertib treatment since the IC_50_ could not be reached, making this cell line potentially useful to understand and explore the mechanisms of resistance to this drug.

We then showed that the addition of Navitoclax to both Ispinesib and Barasertib led to synergistic effects in both cell lines used in this study at concentration levels lower than the IC_50_. Since KSP inhibition leads to prolonged mitotic arrest, we expected the addition of Navitoclax to increase cell death during mitosis. In accordance, we showed here that, in fact, the addition of Navitoclax to Ispinesib exacerbated apoptotic signaling leading to cell death mainly during mitosis. Similarly, since Aurora B inhibition was shown to promote mitotic slippage, we were expecting that the addition of Navitoclax could increase cell death signaling enough to lead to cell death preventing premature mitotic exit. However, the addition of Navitoclax to Barasertib did not significantly affect the number of cells undergoing mitotic slippage but instead increased post-slippage death. Thus, and according to Vorobjev et al.’s model, SCC25 BCL-xL activity, at least after the addition of Navitoclax at the concentration of 3000 nM, should be high enough to still let mitotic slippage occur but not high enough to prevent PSD [24].

Additionally, we showed that the synergistic effects were not exclusive to these drugs but that the inhibition of the same targets with different drugs led to similar results (Figure S1). Furthermore, since one of the most common adverse reactions in clinical trials to the administration of Barasertib is neutropenia, SP-96 could be used as an alternative since it has been suggested that it is able to avoid these types of adverse events [45]. Nonetheless, no clinical trials have been conducted with this inhibitor, and thus, there is a need to further investigate these claims.

Several studies have shown that the inhibition of BCL-2 family members combined with antimitotics leads to increased cell death in several types of cancer [29,30,31,32]. Nonetheless, most have used antimicrotubules, such as Paclitaxel, that as previously stated, have low specificity to cancer cells. Furthermore, as far as we know, our study is the first to test these combinations and approaches in oral cancer cell lines. Moreover, the results presented here are in accordance with those our group has previously reported in non-small cell lung cancer lines with different antimitotics than the ones used in this study, which also showed synergistic effects [32,46]. This study thus contributes to increasing the scientific knowledge regarding this type of approach and further supports its potential for cancer treatment.

In summary, our results showcase the potential therapeutic benefits of concurrently inhibiting BCL-2 prosurvival family members with KSP or Aurora B proteins. Despite the encouraging outcomes observed in the investigated combinations, a constraint within the current study lies in the fact that the in vitro assays were conducted with only two cell lines and that no tests were conducted with these combinations in non-tumoral cell lines to assess if the combinations can improve the selective killing of oral cancer cells while minimizing harm to normal cells, as well as in models that can better mimic the tumor microenvironment such as heterotypic spheroids, and also in animal models. Nonetheless, we intend to perform these experiments in the foreseeable future since further investigations are imperative to gain a deeper comprehension of the underlying mechanisms driving the proposed combinations and to ascertain the in vivo pharmacodynamics and pharmacokinetics.

## 5. Conclusions

In conclusion, the addition of Navitoclax to both Ispinesib and Barasertib demonstrated synergistic effects in oral cancer cells, significantly increasing cell death primarily through enhanced apoptotic activity. Therefore, targeting apoptosis in combination with the inhibitors of KSP and Aurora B could be a more promising strategy to enhance cancer cell killing than using these inhibitors as monotherapy. The data point to a potential anticancer strategy that warrants further exploration.

## Figures and Tables

**Table 1:** IC_50_ values of Navitoclax, Ispinesib, and Barasertib in SCC25 and SCC09 cell lines after 48 h incubation.

	IC_50 (_nM)	
Drugs|Cell Line	SCC25	SCC09
Navitoclax	5197.0 ± 364.0	3754.0 ± 237.0
Ispinesib	3.4 ± 0.5	58.9 ± 3.2
Barasertib	5580.0 ± 664.0	>64,000.0

## Data Availability

The data can be shared upon request.

## Supplementary Materials

The following supporting information can be downloaded at: https://www.mdpi.com/article/10.3390/cancers16112014/s1, Table S1: IC50 values of ABT-737, Filanesib, and SP-96 in the SCC25 cell line, after 48 h incubation; Figure S1: The combinations of ABT-737 with Filanesib or SP-96 in SCC25 showed similar results to those of Navitoclax combined with Ispinesib or Barasertib enhancing cytotoxicity in SCC25. Video S1: Monitoring of an SCC25 cell treated with 1500 nM of Navitoclax that underwent normal cell cycling using timelapse microscopy (DIC). Time in the video is displayed in minutes; available online at https://youtu.be/WCpeu2xA0-0 (accessed on 14 April 2024). Video S2: Monitoring of an SCC25 cell treated with 1.875 nM of Ispinesib undergoing normal cell cycling using timelapse microscopy (DIC). Time in the video is displayed in minutes; available online at https://youtu.be/FTRhFMG3o8g (accessed on 14 April 2024). Video S3: Monitoring of an SCC25 cell treated with 1.875 nM of Ispinesib + 1500 nM of Navitoclax that died during mitosis using timelapse microscopy (DIC). Time in the video is displayed in minutes; available online at https://youtu.be/bJo7rPwBYrU (accessed on 14 April 2024). Video S4: Monitoring of an SCC25 cell treated with 1000 nM of Barasertib undergoing mitotic slippage using timelapse microscopy (DIC). Time in the video is displayed in minutes; available online at https://youtu.be/PjEc6NVpXNk (accessed on 14 April 2024). Video S5: Monitoring of an SCC25 cell treated with 1000 nM of Barasertib + 3000 nM of Navitoclax that died post-slippage using timelapse microscopy (DIC). Time in the video is displayed in minutes; available online at https://youtu.be/8Py7VXCONic (accessed on 14 April 2024). File S1: Original western blots.
