# Supplementary material for: Coupling Kinesin Spindle Protein and Aurora B Inhibition with Apoptosis Induction Enhances Oral Cancer Cell Killing
Source: Cancers (Basel). 2024 May 25;16(11):2014. doi: 10.3390/cancers16112014 (PMC11171144; doi:10.3390/cancers16112014)
Supplement: Supplementary file 1 [file cancers-16-02014-s001.zip › cancers-2992321-supplementary.pdf]

Supplementary file

Table S1. IC_{50} values of ABT-737, Filanesib and SP-96 in the SCC25 cell line, after 48 hours incubation.

**Drugs**	**IC_{50} (nM)**
ABT-737	9,390 ± 440
Filanesib	1.78 ± 0.14
SP-96	4,220 ± 560

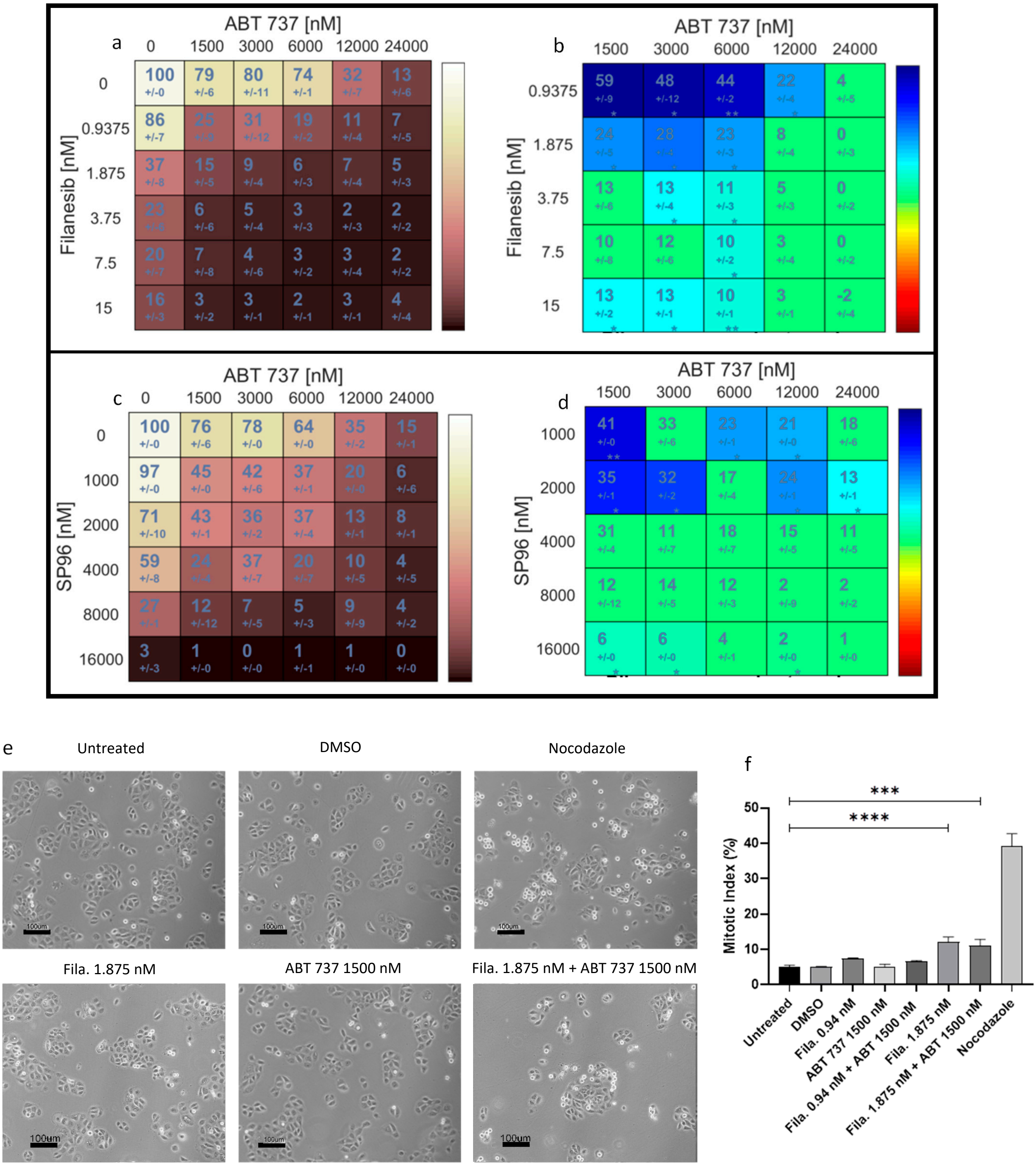
a
ABT 737 [nM]
0 1500 3000 6000 12000 24000
Filanesib [nM]
0: 100 +/-0, 79 +/-6, 80 +/-11, 74 +/-1, 32 +/-7, 13 +/-6
0.9375: 86 +/-7, 25 +/-6, 31 +/-12, 19 +/-2, 11 +/-4, 7 +/-5
1.875: 37 +/-8, 15 +/-5, 9 +/-4, 6 +/-3, 7 +/-4, 5 +/-3
3.75: 23 +/-8, 6 +/-6, 5 +/-4, 3 +/-3, 2 +/-3, 2 +/-2
7.5: 20 +/-7, 7 +/-8, 4 +/-6, 3 +/-2, 3 +/-4, 2 +/-2
15: 16 +/-3, 3 +/-2, 3 +/-1, 2 +/-1, 3 +/-1, 4 +/-4
b
ABT 737 [nM]
1500 3000 6000 12000 24000
Filanesib [nM]
0.9375: 59 +/-9, 48 +/-12, 44 +/-2, 22 +/-4, 4 +/-5
1.875: 24 +/-5, 28 +/-4, 23 +/-3, 8 +/-4, 0 +/-3
3.75: 13 +/-6, 13 +/-4, 11 +/-3, 5 +/-3, 0 +/-2
7.5: 10 +/-8, 12 +/-6, 10 +/-2, 3 +/-4, 0 +/-2
15: 13 +/-2, 13 +/-1, 10 +/-1, 3 +/-1, -2 +/-4
c
ABT 737 [nM]
0 1500 3000 6000 12000 24000
SP96 [nM]
0: 100 +/-0, 76 +/-6, 78 +/-0, 64 +/-0, 35 +/-2, 15 +/-1
1000: 97 +/-0, 45 +/-0, 42 +/-6, 37 +/-1, 20 +/-0, 6 +/-6
2000: 71 +/-10, 43 +/-1, 36 +/-2, 37 +/-4, 13 +/-1, 8 +/-1
4000: 59 +/-8, 24 +/-8, 37 +/-7, 20 +/-7, 10 +/-5, 4 +/-5
8000: 27 +/-1, 12 +/-12, 7 +/-5, 5 +/-3, 9 +/-9, 4 +/-2
16000: 3 +/-3, 1 +/-0, 0 +/-0, 1 +/-1, 1 +/-0, 0 +/-0
d
ABT 737 [nM]
1500 3000 6000 12000 24000
SP96 [nM]
1000: 41 +/-0, 33 +/-6, 23 +/-1, 21 +/-0, 18 +/-6
2000: 35 +/-1, 32 +/-2, 17 +/-4, 24 +/-1, 13 +/-1
4000: 31 +/-4, 11 +/-7, 18 +/-7, 15 +/-5, 11 +/-5
8000: 12 +/-12, 14 +/-5, 12 +/-3, 2 +/-9, 2 +/-2
16000: 6 +/-0, 6 +/-0, 4 +/-1, 2 +/-0, 1 +/-0
e
Untreated
DMSO
Nocodazole
Fila. 1.875 nM
ABT 737 1500 nM
Fila. 1.875 nM + ABT 737 1500 nM
100um
f
Mitotic Index (%)
0 10 20 30 40 50
***
****
Untreated
DMSO
Fila. 0.94 nM
ABT 737 1500 nM
Fila. 0.94 nM + ABT 1500 nM
Fila. 1.875 nM
Fila. 1.875 nM + ABT 1500 nM
Nocodazole

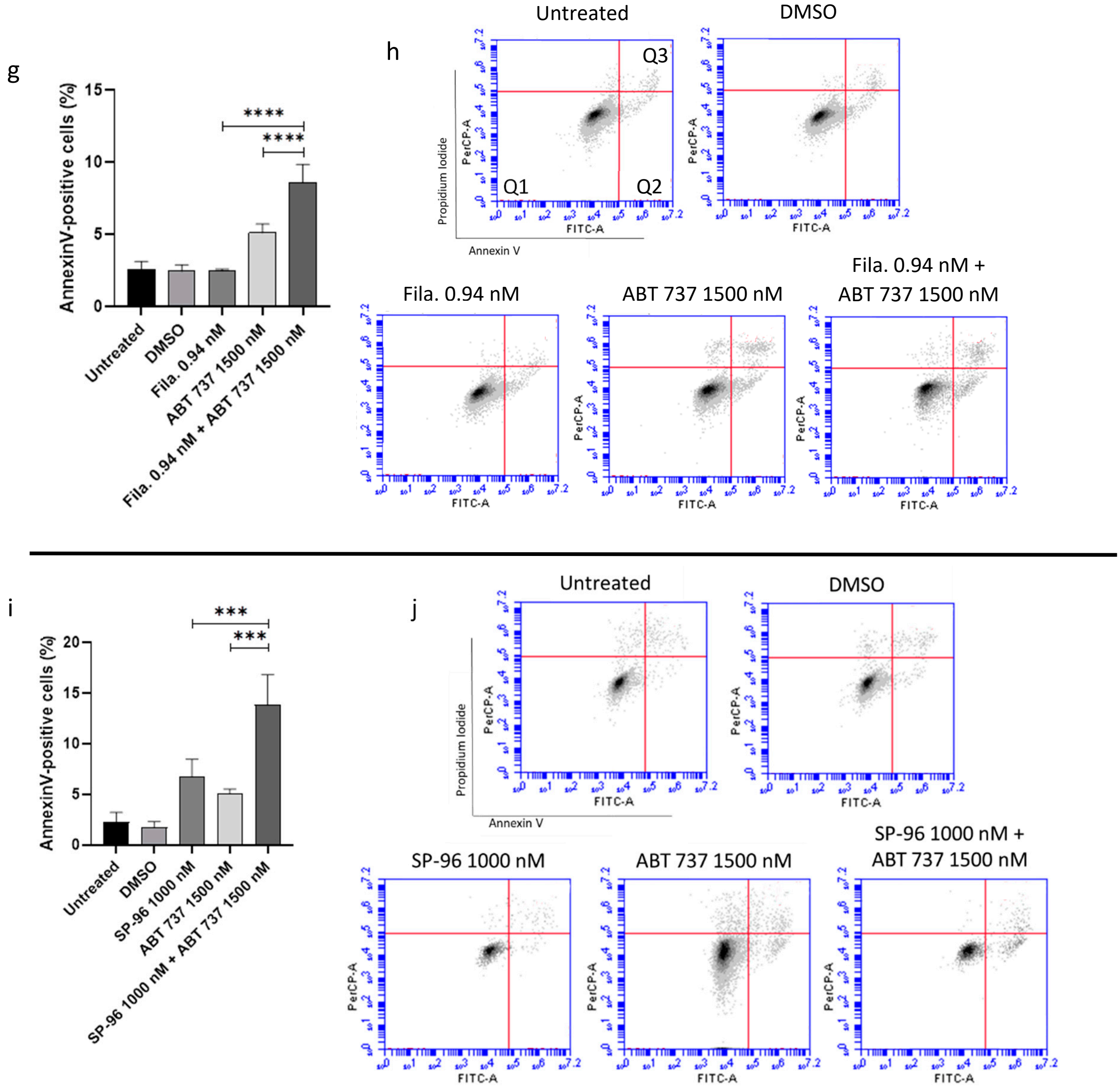

Figure S1. The combinations of ABT-737 with Filanesib or SP-96 in SCC25 showed similar results to those of Navitoclax combined with Ispinesib or Barasertib enhancing cytotoxicity in SCC25. Cell viability (%) following 48 hours of drug exposure both alone or in combination (a,c), assessed by MTT assay with at least three independent experiments. The synergy scores were calculated using the Bliss model of Combenefit software 2.021. Asterisks denote synergistic effects with statistical significance of * $p < 0.05$ and ** $p < 0.01$. (b,d). Filanesib at concentration of 1.875, both alone and in combination with ABT-737 leads to increased mitotic index in oral cancer cells. Representative images acquired by phase-contrast microscopy after drugs exposure for 24 hours for SCC25 (e). Mitotic index quantification for the respective cell lines (f); 0.2 % of DMSO (drug solvent) was used as negative control, while 1 µM of Nocodazole (mitotic blocker drug) was used as positive control. Addition of both Filanesib and SP-96 to ABT-737 increases cell death in the SCC25 cell line. Quantification of Annexin-V-positive cells (g,i). Cytograms demonstrative of oral cancer cell lines double stained with Annexin V-FITC and propidium iodide (PI) (h,j). The quadrants Q are defined as Q1 = living cells (Annexin V- and PI-negative), Q2 = early stage apoptosis (Annexin V-positive/PI-negative), Q3 = late stage apoptosis/secondary necrosis (Annexin V- and PI-positive). The data presented are the average ± standard deviation of three separate experiments. Statistical analysis was performed using one-way ANOVA followed by Tukey's post hoc test for multiple comparisons. *** $p < 0.001$, **** $p < 0.0001$.

Video S1: Monitoring of a SCC25 cell treated with 1500 nM of Navitoclax, that underwent normal cell cycling, using timelapse microscopy (DIC). Time in the video is displayed in minutes; available online at https://youtu.be/WCpeu2xA0-0 (accessed on 14 April 2024).

Video S2: Monitoring of a SCC25 cell treated with 1.875 nM of Ispinesib, undergoing normal cell cycling, using timelapse microscopy (DIC). Time in the video is displayed in minutes; available online at https://youtu.be/FTRhFMG3o8g (accessed on 14 April 2024).

Video S3: Monitoring of a SCC25 cell treated with 1.875 nM of Ispinesib + 1500 nM of Navitoclax, that died during mitosis, using timelapse microscopy (DIC). Time in the video is displayed in minutes; available online at https://youtu.be/bJo7rPwBYrU (accessed on 14 April 2024).

Video S4: Monitoring of a SCC25 cell treated with 1000 nM of Barasertib, undergoing mitotic slippage, using timelapse microscopy (DIC). Time in the video is displayed in minutes; available online at https://youtu.be/PjEc6NVpXNk (accessed on 14 April 2024).

Video S5: Monitoring of a SCC25 cell treated with 1000 nM of Barasertib + 3000 nM of Navitoclax, that died post slippage, using timelapse microscopy (DIC). Time in the video is displayed in minutes; available online at https://youtu.be/8Py7VXCONic (accessed on 14 April 2024).
