# Supplementary figures and images for: Coupling Kinesin Spindle Protein and Aurora B Inhibition with Apoptosis Induction Enhances Oral Cancer Cell Killing
Source: Cancers (Basel). 2024 May 25;16(11):2014. doi: 10.3390/cancers16112014 (PMC11171144; doi:10.3390/cancers16112014)

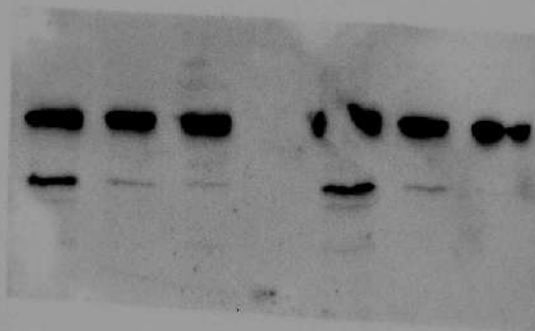

**Aurora B1**

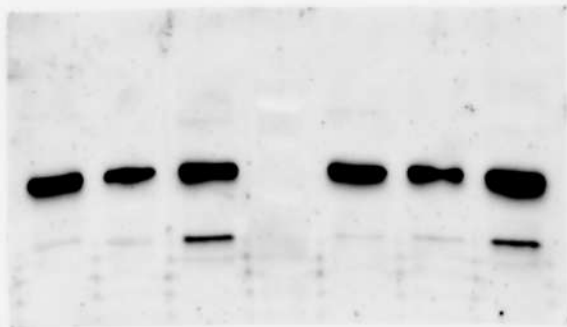

**Aurora B2**

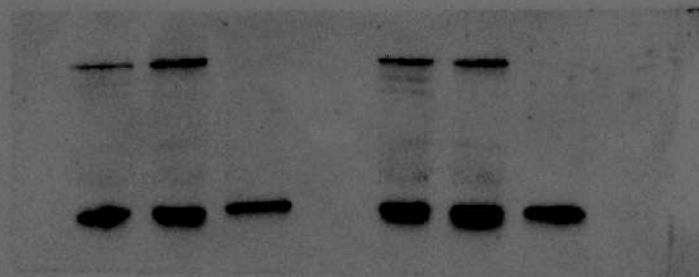

**Eg5 1**

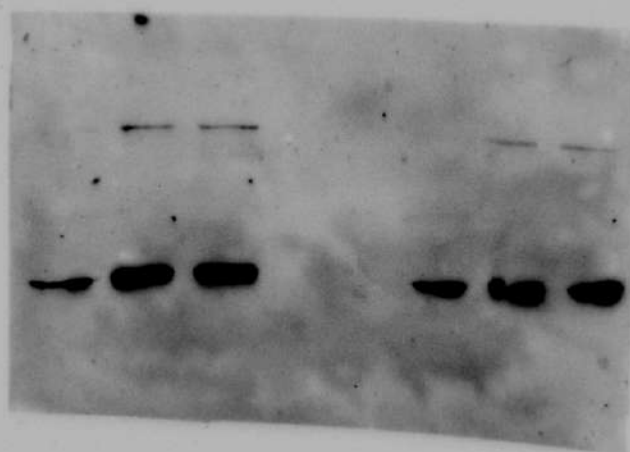

Eg5 2

Supplement: Supplementary file 1 [file cancers-16-02014-s001.zip › cancers-2992321-File S1.pdf]
